# Glycobiology of Human Fungal Pathogens: New Avenues for Drug Development

**DOI:** 10.3390/cells8111348

**Published:** 2019-10-30

**Authors:** Danielle J. Lee, Holly O’Donnell, Françoise H. Routier, Joe Tiralongo, Thomas Haselhorst

**Affiliations:** 1 Institute for Glycomics, Griffith University, Gold Coast Campus, Queensland, 4222, Australia; Member of Fraunhofer International Consortium for Anti-Infective Research (iCAIR), Nikolai-Fuchs Strasse 1, 30625 Hannover, Germany; danielle.lee2@griffithuni.edu.au (D.J.L.); holly.odonnell@griffithuni.edu.au (H.O.); 2 Department of Clinical Biochemistry OE4340, Hannover Medical School, Carl-Neuberg-Strasse 1, 30625 Hannover, Germany; Member of Fraunhofer International Consortium for Anti-Infective Research (iCAIR), Nikolai-Fuchs Strasse 1, 30625 Hannover, Germany; Routier.francoise@mh-hannover.de

**Keywords:** invasive fungal infection, aspergillus, candida, cryptococcus, nucleotide sugar transporter, immunosuppression, GDP-Mannose, UDP-Galactofuranose, UDP-Xylose, UDP-glucuronic acid

## Abstract

Invasive fungal infections (IFI) are an increasing threat to the developing world, with fungal spores being ubiquitous and inhaled every day. Some fungal species are commensal organisms that are part of the normal human microbiota, and, as such, do not pose a threat to the immune system. However, when the natural balance of this association is disturbed or the host’s immune system is compromised, these fungal pathogens overtake the organism, and cause IFI. To understand the invasiveness of these pathogens and to address the growing problem of IFI, it is essential to identify the cellular processes of the invading organism and their virulence. In this review, we will discuss the prevalence and current options available to treat IFI, including recent reports of drug resistance. Nevertheless, the main focus of this review is to describe the glycobiology of human fungal pathogens and how various components of the fungal cell wall, particularly cell wall polysaccharides and glycoconjugates, are involved in fungal pathogenicity, their biosynthesis and how they can be potentially exploited to develop novel antifungal treatment options. We will specifically describe the nucleotide sugar transporters (NSTs) that are important in fungal survival and suggest that the inhibition of fungal NSTs may potentially be useful to prevent the establishment of fungal infections.

## 1. Human Invasive Fungal Pathogens

Fungal infections constitute a broad range of common medical illness from a common superficial or mucosal infection to the more severe systemic Invasive Fungal Infections (IFI) [1,2] that affect millions of people worldwide [3,4]. Fungal infections can occur regardless of the immune status of the host. However, individuals with a compromised immune system are targets for IFI [1,3,5,6,7,8]. The growing number of individuals with compromised immunity is a result of patients with HIV/AIDS, cancer patients undergoing radiation and chemotherapy, hematological conditions, chronic obstructive pulmonary disease, diabetes, and patients receiving immunosuppressant therapy after organ transplant or due to autoimmune disease [1,9,10,11,12,13,14]. The most prevalent pathogenic fungal species that infect these populations are *Cryptococcus, Candida, Aspergillus*, and *Pneumocystis* with reported mortality rates comparable to that of highly monitored infectious diseases such as tuberculosis and malaria [9,15,16,17]. Although most of these are opportunistic fungal pathogens some are commensal species living in the natural human microflora [1,3]. Usually a healthy innate immune system can protect the host from hundreds of potential pathogenic fungal spores daily, with most of these pathogens entering the host via the respiratory system [18,19]. The foreign matter is expelled by the lungs through mucociliary clearance, a primary defense system of the lung, or by the alveolar macrophages that are activated further along the respiratory tract [6,18]. However, in immuno-compromised individuals the ability to fight off these pathogens is either weakened or absent, which can lead to IFI [5]. 

Currently, there is a high rate of morbidity and mortality related to IFI worldwide [1,2,20,21]. There are over 1 billion people affected by some form of fungal infection with over 1.5 million deaths annually [16,17,22]. Despite these high figures, fungal pathogens are heavily under-researched, and the development of treatments and disease surveillance is below that of other comparable microbes such as infectious bacteria [16,17,23,24]. Compared to anti-bacterials, there are only a limited range of antifungals available. This is further restricted by numerous reports of resistance against antifungal drugs such as azoles and echinocandins [24,25,26]. Therefore, there is an urgent medical need to address the growing concern of IFI and the paucity of available treatments. In addition, IFI is a significant economic burden, since it is associated with extended hospital stays leading to high costs for both healthcare systems and patients [27]. The total cost of hospitalization due to aspergillosis and candidiasis in US hospitals from 2005 to 2014 have been estimated with $2.4 billion USD [28]. Despite posing a significant threat to public health, food biosecurity, and biodiversity, fungal infections have remained neglected by governments, pharmaceutical companies, and society for decades and are considered now a serious healthcare issue [9,17,29]. Exploring new potential antifungal drug targets will aid in the discovery and development of novel compounds that will address not only the rising concerns of treatment failures of IFI but also the growing immunocompromised population and economic burden of IFI. 

In this review, the four most prevalent human pathogenic fungal genera, *Aspergillus, Candida, Cryptococcus*, and *Pneumocystis,* will be summarized with a specific emphasis on their prevalence, current treatment options, and their emerging cases of resistance [1]. This review highlights the identification and biosynthesis of various fungal glycoconjugates that contribute to virulence and pathogenicity. Specifically, we will focus on the nucleotide sugar transporters (NSTs) that are essential for the synthesis of these glycoconjugates in opportunistic human pathogenic fungi but may be absent in humans and, therefore, represent attractive potential drug targets to develop novel antifungal treatments.

## 2. Prevalence of Fungal Infections

### 2.1. Aspergillus Genera

*Aspergillus* species are ubiquitous in the environment, found in the air and soil, and live in decaying matter, in stored water facilities, and in bathroom devices [10,18,29]. Pathogenic species from this genus produce airborne spores that, when inhaled by immunocompromised patients, gain access to the alveolar bloodstream and germinate. *Aspergillus* species are the most prevalent pulmonary infection-causing agent in the immunocompromised host [9,13,18,30], with the most invasive and ubiquitous being *Aspergillus fumigatus*, which is followed by *Aspergillus terreus* and *Aspergillus flavus* [18,31]. Fungal infections caused by *Aspergillus* include conditions such as chronic pulmonary aspergillosis (CPA) and allergic bronchopulmonary aspergillosis, which may become invasive, and the often-deadly invasive aspergillosis (IA) [1,13,19]. It has been estimated that more than 300,000 IA cases occur annually worldwide, which leads to a mortality rate of 30–85% depending on treatment [9,16,20,30]. Data collected in the US from 2001 to 2006 found that 20% of all diagnosed IFI cases were IA [24]. CPA is often misdiagnosed as tuberculosis with a high prevalence of about three million cases per year, with approximately a 80% mortality rate within five months if left untreated [9,20].

### 2.2. Candida Genera

*Candida* species are one of the leading fungal pathogens, which cause a range of diseases from common superficial or mucosal infections to invasive infections of the bloodstream [5,6,19]. These commensal species affect both the immunocompromised and immunocompetent patients since they occupy the organs of the gastrointestinal and genital tract, and the largest organ the skin [5,19,32,33]. *Candida albicans* is the most common causative agent with up to 90% of superficial fungal infections caused by this species [10,19,34]. Candidiasis is a very common superficial infection with reports of more than 1 billion people affected worldwide [9]. However, severe infections can arise in an immunodeficient host when the microflora is altered, which results in overgrowth and possible progression into the bloodstream leading to IFI [5,6,33]. Candidemia is a common nosocomial infection with a continually growing mortality rate of 40–50% [10,20,24]. Invasive candidiasis is predicted to increase to more than 700,000 cases annually worldwide [9,16,20]. 

### 2.3. Cryptococcus Genera

*Cryptococcus* species are found globally to colonize soil material [1,6,12,15] and produce easily airborne basidiospores. Like other fungal spores these basidiospores enter the host mainly via inhalation [6,19,23,35]. *Cryptococcus* species are distinct from other opportunistic fungi as their cell wall is enclosed by a polysaccharide capsular layer [5,23,36,37] that not only provides extra protection but functions also as an essential virulence factor. The *Cryptococcus* polysaccharide capsule allows the pathogen to spread and infect the brain, which can result in high mortality rates [23,37]. Cryptococcosis generally develops from infection with *Cryptococcus neoformans* and can progress from pneumonia to meningitis in immunocompromised patients [1,4,5,35]. Cryptococcal meningitis is caused when the fungal cells invade the bloodstream or the lymphatic system with selectivity for the central nervous system [1,4,15,19]. About 220,000 cases of Cryptococcal meningitis are reported annually, with a mortality rate ranging from 10–70% depending on treatment and geographical location of the patient [20,37,38]. An additional life-threatening species is *Cryptococcus gattii*, which can cause IFI in both the immunosuppressed and immunocompetent hosts [1,12,15,24]. More than 957,000 cases of meningoencephalitis in HIV patients caused by *Cryptococcus* have been identified, leading to 624,700 patient deaths [19].

### 2.4. Pneumocystis Genera

The fungal genus *Pneumocystis* consists of four species with the most prevalent pathogenic species being *Pneumocystis jirovecii*. *P. jirovecii* causes pneumocystis pneumonia, which is an opportunistic infection that is particularly common in immunocompromised patients. *Pneumocystis jirovecii* infections have been predicted to surpass 400,000 cases per year and the mortality rate for this infection varies from 13% to 80% depending on a variety of factors, including treatment [15,20,24,39]. Although the worldwide mortality statistics of *P. jirovecii* were reduced by approximately 10% with the introduction of effective antiretroviral therapy for HIV patients, *P. jirovecii* is still one of the main causes of death in this immunocompromised population [1,9,39]. Unlike other fungal pathogens, *Pneumocystis* species lack a broad range of fundamental functions such as lacking the capacity for lipid metabolism, the absence of ergosterol synthesis, and a deficiency in genes required for amino acid synthesis, and such are heavily dependent on the host [19]. 

## 3. Surveillance of IFI

Given the high prevalence of IFI, surveillance of these highly invasive human pathogens remains inadequate but is unequivocally required to provide a comprehensive systematic review of the worldwide burden of IFI and the effectiveness of the current treatment regimens [24]. Currently, there is no international body (including the World Health Organization, WHO) that collects standardized data on the burden caused by these pathogens [9,24,40]. The only available information originates from individual facility-based studies that are limited in providing standardized data since they are usually collected from hospital records [9,24]. The US Center for Disease Control and Prevention (CDC) is the only government organization that performs any monitoring of IFI [15].

## 4. Diagnosis of IFI

Early detection of IFI is of paramount importance to improve patient outcomes but currently available diagnostic methods often involve invasive procedures or cultures that may lack sensitivity, which leads to diagnosis difficulties and inaccuracies [14,24,41,42]. Furthermore, this invasive process can be slow and challenging in immunocompromised patients due to their underlying condition and the requirement for a sterile sample [43,44,45,46]. The gold standard method for diagnosis involves bronchoalveolar lavages or lung biopsies that are then tested by histological examination [10,41,45,47]. Novel non-invasive diagnostic tools have been developed, including polymerase chain reaction-based tests, β-glucan assays, and a monoclonal antibody immunoassay, which may be useful for certain clinical groups [39,42,45,47]. Galactomannan antigen tests for detecting IFI are also available but show possible cross-reactivity to antibiotics the patient may be taking (e.g., β-lactam antibiotics) [14]. 

## 5. The Current Treatment of IFI and the Development of Resistance 

### 5.1. Azoles

**Figure 1:**
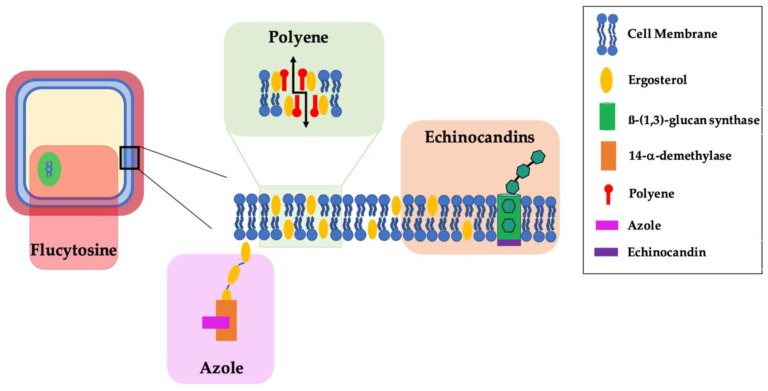
Site of action of currently available antifungal treatments.

Azoles are a class of broad-spectrum antifungals that inhibit lanosterol 14-α-demethylase, which is the enzyme that synthesizes ergosterol from lanosterol (Figure 1) [8,13,48]. Ergosterol is a major component of the fungal plasma membrane, which controls the cell integrity and the functioning of transmembrane enzymes [2]. The disturbance of sterol leads to cell membrane instability and toxicity due to build-up of the precursor lanosterol [2,4,13,49]. Lanosterol 14-α-demethylase is a cytochrome P450 (CYP450) dependent enzyme [4,13,31,49]. Azoles are classified into two categories: (i) imidazoles for compounds that contain two nitrogen atoms in a five-membered heterocyclic ring with a halogenated phenyl group and (ii) triazoles if three nitrogen atoms are present [1,31]. Resistance to azoles is well documented due to their widespread use as prophylaxis, and due to their use in veterinary applications and agriculture where antifungals are used to protect crops and timber [8,48,49,50]. The most reported mechanism of resistance is through overexpression of genes encoding the lanosterol 14-α-demethylase enzyme [2,4,13]. Other mechanisms are mutations in the gene, which cause a structural change near the active site that hinders azole binding [4,13,31] and upregulation of multidrug transporters such as ATP-binding cassette transporters [4,13]. Azoles form substrates to this transporter due to the close homology, which increases the efflux of the azoles [4,13]. 

Another major concern is the high probability of drug-drug interactions because many immunocompromised patients take several other drugs such as immunosuppressants on a daily basis to treat their underlying condition [49]. These drug interactions occur because of the non-selective binding of the azole to the mammalian CYP450 enzyme, which is involved in the metabolism of many drugs [49]. For example, CYP450-dependent triazoles (especially itraconazole and voriconazole) cause adverse reactions when administered with other drugs such as statins, warfarin, and tacrolimus [14,15,48,49]. Therefore, co-administration of these drugs is contraindicated or requires adjustment of dose and strict monitoring [14]. This can further restrict already limited treatment options, which is especially concerning in the case of voriconazole, which is considered as the first line of treatment for many fungal infections [14]. Nevertheless, the current treatment of choice against IA remains voriconazole or isavuconazole [45,51].

### 5.2. Polyene

Polyenes act by binding ergosterol in the fungal cell membrane, which forms polyene-ergosterol complexes that lead to membrane-spanning channels and subsequent leakage (Figure 1) [8,48,49,52]. This increased permeability gives rise to changes in the cell’s composition and causes the exchange of small molecules, which results in oxidative stress and cell death [1,2,31]. Amphotericin B (Amp B) is the most frequently used polyene derivative that has been successfully administrated to treat IFI [1,31]. However, the use of Amp B is limited due to bioavailability, solubility, and dose-related toxicity of Amp B [1,8,15,50]. In order to address these limitations, lipid formulations have been developed [8,48,49].

### 5.3. Echinocandins

Echinocandins are inhibitors of the β-(1,3)-glucan synthases, which is an enzyme involved in the biosynthesis of the fungal cell wall (Figure 1) [7,26,49,52]. Inhibition of this enzyme leads to a decrease in β-(1,3)-glucans production, which makes up the backbone of the cell wall. This consequently leads to diminished integrity of the cell wall, which allows the fungal cell to be exposed to external stress factors and osmosis, and leads to rapid cell death [2,4,31]. Echinocandins are cyclic hexapeptides, with each different echinocandin having a different substituent at the fifth carbon of the ring [2,31]. Echinocandins have a well-established favorable safety profile due to the absence of β-(1,3)-glucan and β-(1,3)-glucan synthases in mammalian cells [2]. The rise of resistance is largely due to the increased prophylactic use of echinocandins and the existence of biofilms within the host. The biofilm provides an environment ripe for the development of drug resistance, and, subsequently, acts as an endogenous reservoir for resistant mutants [7,31,49]. 

An amino acid sequence mutation in the conserved regions of the genes *fks1* or *fks2* have been described. These genes are fundamental since they encode the highly active subunit of the glucan synthase [2,4,7,25]. A poor response to echinocandins has been reported with *fks* mutants of *C. albicans* and *C. glabrata* [7,26]. Additional modes of resistance include the induction of adaptive protection mechanisms, which create persister cells resistant to echinocandins [7]. This has also been observed in *A. fumigatus* and *C. albicans* in combination with the cell’s ‘paradoxical effect’ or mode of interdependence between chitin and β-(1,3)-glucans [7,25,26]. It manifests as an increase in chitin content in the cell wall to compensate for β-(1,3)-glucan loss [11,25,31,52]. Echinocandins are still recommended as the drug of choice in most cases for treating invasive candidiasis [53]. In confirmed cases of resistance, lipid formulation of Amp B is recommended [53].

### 5.4. Pyrimidine Analogue Flucytosine

Flucytosine is a pyrimidine analogue nucleic acid synthesis inhibitor (Figure 1) [2,25,48,52]. Access to the fungal cell occurs via the transport protein cytosine permease, before deamination by cytosine deaminase to 5-fluorouracil (5-FU) [2]. 5-FU is transformed into 5-fluorouridine monophosphate before phosphorylation and incorporation into the RNA synthesis instead of uridine triphosphate [2]. This process interferes with the RNA synthesis and, consequently with protein synthesis. Another mechanism of action is by forming 5-fluorodeoxyuridine monophosphate, which inhibits the synthesis of thymidine required for DNA biosynthesis [2]. Flucytosine has shown selectivity for fungal cells due to the absence of cytosine deaminase in human cells [2]. The activity of flucytosine is limited to some strains of yeast such as *Candida* or *Cryptococcus* and there are reports of resistance to 5-FU and, therefore, adjunct therapy is commonly seen [2].

## 6. The Glycobiology of the Fungal Cell

### 6.1. Cell Membrane

The cell membrane is a structure required for many essential processes including regulation of vesicles in and out of the cell, uptake of nutrients, biosynthesis of the cell wall, and regulation of morphological changes [33]. A major component of the cell membrane is ergosterol, which is a sterol present in fungal cells that plays an equivalent role to cholesterol in humans. This provides flexibility while keeping the membrane stable [2]. Another fungal specific plasma membrane constituent is inositolphosphoceramides such as glycosylinositolphosphoceramides (GIPCs), which is a sphingolipid and important for fungal growth and interaction with the host [54]. The cell membrane houses the enzyme, lanosterol 14-α-demethylase (the target for azoles, see Section 5.1), and transmembrane enzymatic complexes that are crucial in the biosynthesis of the cell wall constituents [2,48]. It also has great structural importance since it forms the bases to which glycosylphosphatidylinositol (GPI) is inserted [55,56,57,58].

**Figure 2:**
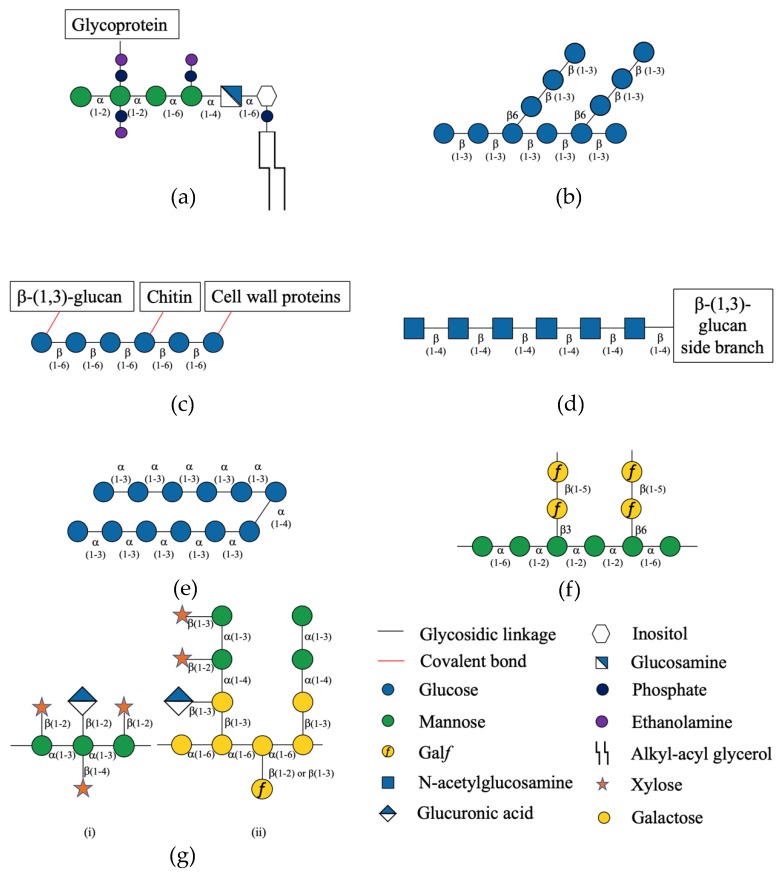
Schematic structure of (**a**) Glycosylphosphatidylinositol (GPI), and common fungal polysaccharides (**b**) β-(1,3)-glucan, (**c**) β-(1,6)-glucan, (**d**) Chitin, (**e**) α-(1,3)-glucan, (**f**) Galactomannan, found in *Aspergillus* genera, (**g**) (i) Glucuronoxylomannan (GXM), (ii) Galactoxylomannan (GXMGal), found in the capsule of *Cryptococcus* genera.

GPI is a glycolipid on the cell membrane that plays an important role in cell wall biosynthesis and cell morphology as a carrier of cell surface proteins [25,59,60,61]. GPI is synthesized in the endoplasmic reticulum and consists of mannose (Man) residues, a glucosamine, a phospholipid, and inositol making up its backbone with ethanolamine phosphate side chains (Figure 2a) [55,56,57,60]. Further modification is then carried out in the Golgi lumen [62]. The anchoring of the cell surface proteins to the cell membrane by GPI enables them to perform post-translational modification [55,56,57,58]. For example, the *A. fumigatus* glucanosyltransferase (Gel1), which is a GPI-anchored enzyme, is involved in the elongation of linear β-(1,3)-glucans and the formation of β-(1,3)-glucans branching [59,61,63,64]. Incorporation and cross linking of β-(1,3)-glucan with chitin and galactomannan is also performed by a GPI-anchored protein (Dfg family) [59,61,64]. Furthermore, GPI-mannoproteins in *Candida* bind to the β-(1,6)-glucans in the cell wall, which enable them to localize to the cell surface and become available to act on external matrixes [11,35,57,60]. 

### 6.2. The Fungal Cell Wall

The cell wall is a distinctive component of the fungal pathogen that is absent in mammalian cells [5,31,65,66]. It protects the fungal cell from external factors and enables the pathogen-host contact [21,23,31,67]. The cell wall is a dynamic, complex structure that is both flexible and relatively rigid [31,61,65,66]. This rigidity allows it to withstand turgor pressure while remaining malleable enough to allow the cell morphological modification as a result of external triggers to grow [21,32,59,66]. Nevertheless, it is the rigidity that assists the cell’s penetration of the host tissue, which allows the fungal pathogen to establish infection [61,66]. This property of the fungal cell is due to its polysaccharide composition, which makes up 90% of the cell wall [11,61].

The cell wall can be divided into two sub-layers with the inner layer conserved between different species [66]. The conserved inner layer is made up of β-(1,3)-glucan, and chitins covalently linked to other polysaccharides such as β-(1,6)-glucan or galactomannan [21,23,32,68]. On the other hand, the outer layer of the cell wall is heterogenous in its structure with significant variability between species. It includes α-(1,3)-glucan, galactomannan, mannan, glycosylated proteins, melanin, which can also be found in the inner layer in some species, and galactosaminogalactan [21,23,59,67,68]. The constituents of the outer layer form the basis of its adherent properties when infecting the host while also protecting it from phagocytosis of the host immune response [25,32]. In *Cryptococcus* genera, the cell wall is highly associated with the formation and structure of the cell capsule, which surrounds the cell wall [23]. Alteration or disturbance of the cell wall can lead to exposure of the cell membrane, which results in rupture and lysis due to external stress factors [25].

#### 6.2.1. β-(1,3)-Glucan 

β-(1,3)-glucans, which compose about 50% of the cell wall [2], are made up of repeating glucose (Glc) residues linked through β-(1,3) linkages (Figure 2b) [61,65]. They are synthesized by the glucan synthase complex that is located in the cell membrane [31,61,63,65,66,68,69]. The catalytic subunit of this transmembrane complex consists of up to 16 transmembrane helices, depending on the species [61,66,68,70]. The glucan synthase complex uses the nucleotide sugar UDP-Glc to produce a growing linear glucan polysaccharide [25,66,70]. This structure is then released in the periplasmic space where it is branched by glucanosyltransferases and can hydrogen bond with other polysaccharides, such as chitin, to form the cell wall skeleton [31,32,65,66]. Elongation and remodeling of the polysaccharides’ three-dimensional network is required for hyphal growth and occurs as a response to external stress [31].

The catalytic subunit of the β-(1,3)-glucan synthase complex (the target for echinocandins, see Section 5.3) is encoded by diverse genes depending on fungal species, with their deletion impacting the cell viability [31,61,70]. Both *A. fumigatus* and *C. neoformans* express a single catalytic subunit encoded by the *fks1* gene [23,31,65]. Deletion of *fks1* in *Aspergillus* results in a characteristic growth defect phenotype similar to the effect of echinocandin treatment in wild type *Aspergillus* [31,71]. In contrast, a lethal phenotype was observed when *fks1* was inactivated in *C. neoformans* [23]. Similarly, in *Saccharomyces cerevisiae*, which has two *fks* genes, mutations of both *fks1* and *fks*2 led to cell death [25,61]. Elongation and inter-networking of β-(1,3)-glucans in the periplasmic space by the β-(1,3)-glucanosyltransferases is also required for fungal growth. Of the seven different genes that encode β-(1,3)-glucanosyltransferase in *A. fumigatus*, only one gene, *gel4*, was essential for the fungus [31]. Gel4 plays an important role in both the elongation and branching of β-(1,3)-glucan, which verifies the essentiality of this enzyme in the construction of the cell wall [72].

#### 6.2.2. β-(1,6)-Glucan 

β-(1,6)-glucan is another significant component of the fungal cell wall, especially in *C. neoformans* due to its effect on the organization of the polysaccharide capsule [23]. The function of β-(1,6)-glucan in species such as *S. cerevisiae* and *C. albicans*, is related to the organization of the cell wall through interactions with other cell wall constituents such as β-(1,3)-glucan, chitin, and cell wall proteins (Figure 2c) [23,63]. Despite its importance, the biosynthesis of β-(1,6)-glucans is yet to be elucidated [23,25]. Several genes have been suggested to be implicated in β-(1,6)-glucan biosynthesis (*kre5*, *kre6*, and *skn1*) [23,25]. *Cryptococcus* species become avirulent when *kre5* is mutated or when *kre6* and *skn1* are deleted [23,66]. This phenotype is linked to a change in cell wall integrity, exposure of the cell to osmotic and environmental stress, and an altered cell capsule [23]. Therefore, the biosynthesis of β-(1,6)-glucan plays an essential role in *Cryptococcus’* survival through cell wall integrity and cell capsule biosynthesis [23].

#### 6.2.3. Chitin 

Chitin is a linear polysaccharide of β-(1,4)-*N*-acetylglucosamine (GlcNAc) that acts as the scaffold for the fungal cell wall (Figure 2d) [23,61,66,70]. Chitin provides the cell wall with structure and strength by forming covalent bonds with other cell wall constituents [11,23,32,35]. Chitin synthases are transmembrane enzymatic complexes in the plasma membrane that utilize UDP-GlcNAc as donor substrates to form the linear β-(1,4)-linked chain required for the cell wall [63,65,68,69]. Depending on the fungal species, the number of genes encoding these synthases can vary [61,66]. The deacetylated form of chitin, chitosan, is a more flexible polysaccharide with higher solubility. It is found in the phylum of ascomycetes, zygomycetes, and basidiomycete [23,61,66,69]. Chitosan replaces chitin in the cell wall and is likewise important, with chitin deacetylase knockouts exhibiting increased sensitivity to cell wall stress [23,35,73].

The number of genes encoding the chitin synthase can vary from 1-20 depending on the fungal species, even though they are not all essential for virulence [25,61]. Chitin has been extensively studied in *S. cerevisiae*, with three synthases involved in its biosynthesis, which include Chs1, Chs2, and Chs3 [25,61]. *A. fumigatus, A. nidulans*, and *C. neoformans* have eight chitin synthases [23,31,68,70] while *C. albicans* has four [25]. Single gene mutations are not lethal, but double chitin synthase knockouts are lethal in *Aspergillus* species [66]. The double deletion of *csmA* and *csmB* that encode two of the eight chitin synthases in *A. nidulans* was shown to be lethal while single mutation caused a reduction in the chitin and an altered cell phenotype [31,74]. Chitin synthases are therefore essential in the formation of the cell wall and with their absence in mammalian cells, show potential as antifungal drug target [2]. Chitin also plays a role in stimuli reactions such as the compensatory ‘paradoxical’ effect (see Section 5.3), which is important for understanding the rise of antifungal drug resistance [7].

#### 6.2.4. α-(1,3)-Glucan 

**Figure 3:**
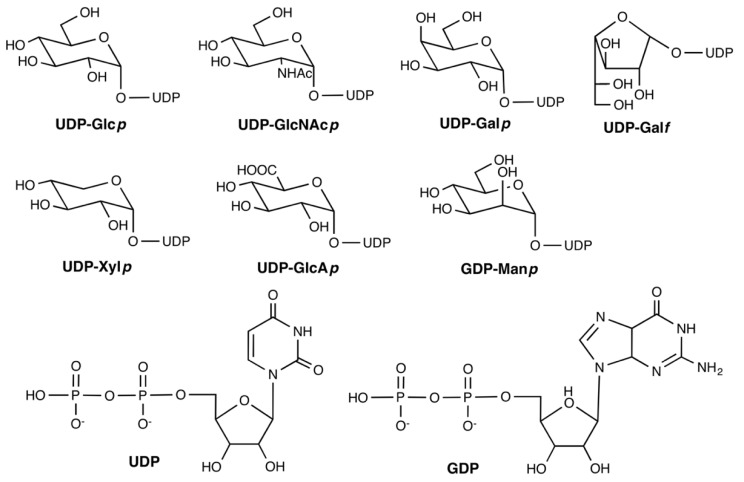
Chemical structures of important nucleotide sugars: UDP-Glc*p*, UDP-GlcNAc*p*, UDP-Gal*p*, UDP-Gal*f*, UDP-Xyl*p*, UDP-GlcA*p*, and GDP-Manp and nucleotides diphosphate UDP and GDP. *Abbreviations*: UDP (Uridine diphosphate), GDP (Guanosine diphosphate), Glc (Glucose), Gal (Galactose), Xyl (Xylose), GlcA (Glucuronic acid), GlcNAc (*N*-acetylglucosamine), *p* (pyranose), and *f* (furanose).

α-(1,3)-glucans (Figure 2e) are generally a minor component of most fungal cell walls, which is completely absent in *Candida* [66]. An exception to this includes the *Cryptococcus* species, where the cell wall possesses relatively high levels of α-(1,3)-glucan that link the cell wall to the polysaccharide capsule [35,66,69]. These glucans are synthesized by the corresponding transmembrane α-(1,3)-glucan synthases using UDP-Glc (see Figure 3 for structure) as the donor substrate [25,35,69]. Depending on the species, the number of α-(1,3)-glucan synthase encoding genes varies [59]. Deletion of *ags1* in *A. fumigatus* only reduced α-(1,3)-glucans in the cell wall by approximately 50% [75]. The successive deletion of *A. fumigatus ags1*, *ags2*, and *ags3* genes led to an absence in α-(1,3)-glucans, which shows a redundancy of the three α-(1,3)-glucan synthases. Absence of α-(1,3)-glucans was dispensable for growth but did affect virulence due to structural changes in the cell wall, which leads to increased killing by phagocytes [75,76]. In *C. neoformans*, inhibition of α-(1,3)-glucans biosynthesis by RNA interference interrupted capsule biosynthesis, abrogated growth at 37 °C, which reduced virulence [25,70,77]. In all mutants, the distribution of cell wall constituents such as β-glucan and chitin compensate for the loss of α-(1,3)-glucans and the cell is still malformed [35,70]. Therefore, targeting the α-(1,3)-glucan biosynthesis is also an excellent opportunity for antifungal drug discovery, since it has the ability to alter the integrity of the cell wall leading to cell lysis.

#### 6.2.5. Galactomannan 

The polysaccharide galactomannan is an essential part of the cell wall in *Aspergillus* species, consisting of a linear α-mannose (Man) main core (through α-(1,2) and α-(1,6) linkages) with β-(1,3), β-(1,6), and β-(1,2) branched galactose (Gal) moieties (Gal*f*, Figure 3) [65,67,68,70,78]. The galactomannan found in the fungal cell wall is covalently bound to β-(1,3)-glucans, which are secreted in the extracellular matrix or attached to the cell membrane by GPI-anchor forming lipogalactomannan [63,67,68].

**Figure 4:**
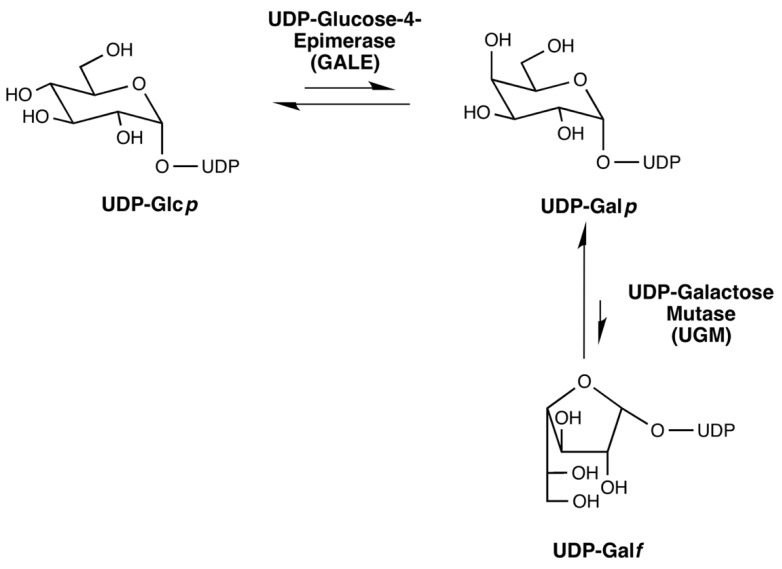
Biosynthesis of UDP-Gal*f*.

In mammalian cells, Gal is only present as galactopyranose (Gal*p*) in the form of a six-membered ring (five carbon atoms and one oxygen atom) [67,79]. In fungi, however, Gal is also found as galactofuranose (Gal*f*), which is a five-membered antigenic ring [42,67,79,80]. The biosynthesis of Gal*f*-containing glycoconjugates begins with the synthesis of UDP-Gal*f,* which is obtained from UDP-Gal*p* by the microbe specific UDP-Gal*p* mutase (UGM), present in the cytosol (Figure 4) [81,82]. The encoding gene (*glf* or *ugm*) has been found in fungi that belong to the subphylum of Pezizomycotina in the class of Ascomycota, and in *C. neoformans* [67]. Incorporation of Gal*f* into relevant glycoconjugates occurs in the Golgi apparatus [63,83,84]. Deletion of the gene encoding UGM (g*lfA)*, the UDP-Galactofuranose transporter (g*lfB)*, or the galactofuranosyltransferase GfsA in *A. fumigatus*, led to an absence of a galactofuran side chain of galactomannan and was associated with a growth impairment [63,85,86,87]. Similarly, deletion of the galactosyltransferase genes *gfsA*, *gfsB*, and *gfsC* in *A. niger*, demonstrated their implication in galactomannan biosynthesis and the redundancy of GfsA and GfsC in galactofuranosylation [88]. The main linear α-mannose (Man) main core structure is synthesized from the precursor GDP-Man and deletion of the GDP-Man transporter (GMT) resulted in the absence of galactomannan, which confirmed that this polysaccharide is synthesized in the Golgi and this GMT deletion was associated with a severe growth defect [63]. Two newly discovered α-(1,2)-mannosyltransferases encoded by *cmsA/ktr4* and *cmsB/ktr7* have been found to be essential for galactomannan biosynthesis, with their absence leading to reduced virulence of *Aspergillus* in a mouse model of IA [89,90]. In contrast, these genes in yeast code proteins involved in glycosylation [89,90].

### 6.3. Cell Capsule: Cryptococcus Polysaccharide Coating

In addition to a protective cell wall, *Cryptococcus* species have a polysaccharide capsule, [32,35,69,91] which is an important *Cryptococcus* virulence factor [23,36,37,69]. The polysaccharide capsule allows the cell to survive in the human host, as well as facilitate an interaction with the host’s endothelial cells. This allows the fungal cells to adhere and cause an infection [1,23,35]. This extra layer is formed outside the cell wall when the fungal pathogen is exposed to conditions that represent a host or by induced stress [23,91]. Just like the cell wall, the capsule has a dynamic structure [35]. Depending on the site of infection, environmental factors trigger the capsule to change size and thickness [1,23,35]. This virulence factor is highlighted by the cell capsule having the ability to expand in size, which protects the cell from phagocytosis associated with the host immune response [1,35]. 

The cell capsule is synthesized with two main polysaccharides, glucuronoxylomannan (GXM) and galactoxylomannan (GXMGal) [32,36,37,69]. GXM is a large polysaccharide composed of a linear α-(1,3)-Man backbone with a side branch of glucuronic acid (GlcA) through β-(1,2) linkage to the Man and Xylose (Xyl) residue via either β-(1,2) or (1,4) linkage depending on the strain (Figure 2g(i)) [35,69,92]. It comprises about 90% of the total cell capsule weight [23,35,66,69]. The smaller constituent of the capsule, GXMGal, has a linear α-(1,6)-Gal backbone (which is sometimes substituted for β-(1,2) or β-(1,3) Gal*f* at an unbranched Gal residue) with side branching of Man and Gal with variable substitution of GlcA and Xyl (Figure 2g(ii)) [36,37,69,92,93]. GlcA is a fundamental sugar residue that appears to be required only for the biosynthesis of GXM and GXMGal in *C. neoformans*, aside from its role as a precursor to UDP-Xyl [37]. UDP-Xyl is produced from UDP-GlcA through a decarboxylation reaction catalyzed by UDP-glucuronic acid decarboxylase enzyme [35,37]. Figure 3 shows the chemical structure of the nucleotide sugars UDP-GlcA and UDP-Xyl.

### 6.4. Other Glycans Found in Fungi

In addition to the predominant polysaccharides already mentioned in this review, there are a number of other polysaccharides and glycoconjugates, including some of which possess important functions while, for others, little is known. These include mannoproteins (or cell wall proteins), galactosaminogalactan (GAG), and mixed linkage β-(1,3)/(1,4)-glucans. Mannoproteins are comprised of mannans covalently attached through *N*-linkages or *O*-linkages to proteins on the cell wall [67]. The *O*-linked glycans are short chains of α-(1,2)- or α-(1,3)-mannoses while the *N*-linked glycans may carry up to 200 mannose residues (linear α-(1,6) residues with side branching of α-(1,2) or α-(1,3)-Man attached to two GlcNAc residues) [11,32,66,94]. The biosynthetic pathway of these structures varies, and, in *Aspergillus* species, they contain Gal*f* on the non-reducing end of the Man backbone [67]. Therefore, alteration to Man or Gal*f* production affects this glycosylation process [86,95]. 

Sialic acids are a large family of neuraminic acid derivatives with a nine-carbon backbone. More than 50 different sialic acid forms have been identified in nature, including the most abundant *N*-acetylneuraminic acid (Neu5Ac). Sialic acids are typically found at the ends of glycans decorating the cell-surface of vertebrates and higher invertebrates, and play important roles in many aspects of vertebrate physiology, from intercellular adhesion and signaling, to microbial attachment [96]. The expression of sialic acid in several pathogenic fungal species has been suggested by a number of groups including in *Cryptococcus neoformans* [97], *Candida albicans* [98], *Fonsacaea pedrosoi* [99], *Paracoccidioides brasiliensis* [100], and *Aspergillus fumigatus* [101,102]. The role of sialic acid in infection is unclear, even though Wasylnka et al., in 2001, found that pathogenic species of *Aspergillus* tend to have a higher concentration of sialic acids on the surface of their conidia [103]. 

GAG is a polysaccharide composed of galactosamine, α-(1,4) linked galactose and *N*-acetylgalactosamine [104,105] residues found on the cell membrane of *Aspergillus* and has been shown to contribute to pathogenesis leading to IA [104,105]. This polysaccharide has an important function in regulating the adherence of the pathogenic cell in the host as well as in the production of a biofilm [104,105]. Mixed linkage β-(1,3)/(1,4)-glucans are another polysaccharide found within the *Aspergillus* cell wall mesh work along with β-(1,3)-glucan, galactomannan, and chitin [106]. In *Aspergillus*, the biosynthesis and role of galactosaminogalactan is still unknown, with studies predominantly conducted in plants [106]. However, Samar et al. in 2015 identified a β-(1,3)/(1,4)-glucan synthase, named ‘Three Four Transferase 1′ or *tft1* that influences the level of the mixed linkage glucan in *Aspergillus* [106]. More studies are needed to further understand the role and function of mixed linkage β-(1,3)/(1,4)-glucans.

## 7. Nucleotide Sugar Transporters: Door to Fungal Virulence

**Figure 5:**
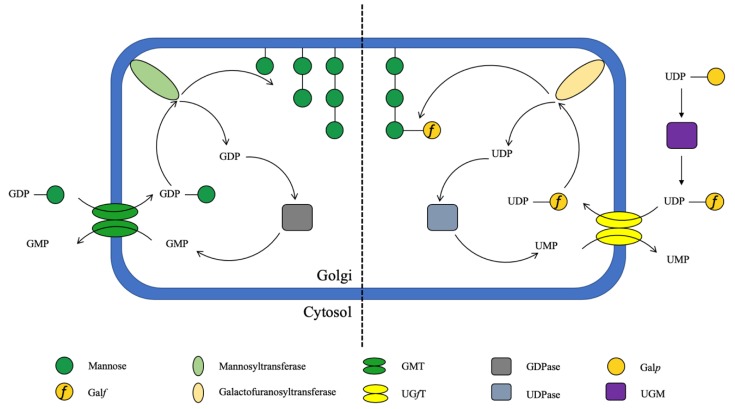
Schematic of the role of NSTs in the formation of mannans and galactomannans. See Figure 3 for chemical structures.

The biosynthesis of several important cell wall-associated polysaccharides and glycoconjugates occurs in the secretory pathway, catalyzed by glycosyltransferases located in the Endoplasmic Reticulum (ER) and/or Golgi apparatus lumen, using dolichol-phosphate activated monosaccharides or nucleotide sugars as the donor substrates [107,108,109,110,111,112]. Nucleotide sugar substrates are usually synthesized in the cytosol before translocation into the Golgi and/or ER lumen [110,113,114,115]. To facilitate this translocation, NSTs, which are a family of solute transporters, are required [107,109,113,116]. These transporters function as antiporters, where the translocation of cytosolic nucleotide sugars into the Golgi and/or ER is coupled with the export of the corresponding nucleotide monophosphate (NMP) (Figure 5) [111,114,115,117]. As previously outlined in this review, mutation or knockdown of fungal genes encoding NSTs results in specific polysaccharide deficiencies (i.e., Galactomannan) [107,109] with a strong impact on fungal growth, virulence and fungal survival. As such, fungal NSTs may represent excellent drug targets for the development of novel treatment options.

### 7.1. GDP-Mannose Transporter (GMT)

Mannose (Man) is one of the most abundant sugars present in the fungal cell [36]. For the biosynthesis of a Man-containing polysaccharide including galactomannan, mannoproteins and previously mentioned biosynthesis of the *Cryptococcal* cell capsule [63], GDP-Man must be transported, from the cytosol into the Golgi by the GDP-Man transporter (GMT) (Figure 5) [112,118]. 

The key role played by the GMT in the biosynthesis of the fungal galactomannan cell wall was highlighted by the absence of galactomannan synthesis following the targeted deletion or mutagenesis of the GMT [3,63,119]. A GMT mutant in *A. fumigatus* showed severely decreased growth with signs of altered morphology due to alteration of the cell wall [63]. An analysis of the cell wall composition of the null mutated *gmtA* compared to the wild type revealed a lack of Man, which is consistent with the cell’s deficiency in galactomannan [63]. Mutation of the GMT in the yeasts *S. cerevisiae*, *C. albicans*, *C. glabrata*, and in *A. nidulans* has been found to alter the production of mannosylated glycoproteins and, hence, led to defects in growth and shape, with reduced Man content and increased drug sensitivity [3,117,119]. 

**Figure 6:**
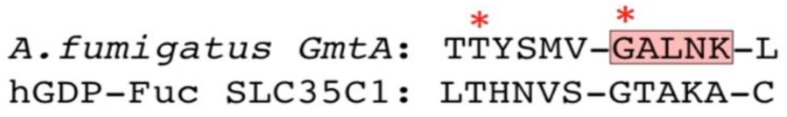
The essential GALNK-motif in the *A. fumigatus gmtA* (GDP-Man transporter, GMT) is not conserved in the human GDP-fucose transporter (GFT, SLC35C1).

Recently, our structural understanding of the GMT has increased significantly due to elucidation of the x-ray crystal structure of the yeast GMT Vrg4 (Vanadate resistance glycosylation protein 4) by Parker and Newstead [110]. The x-ray crystal structure of Vrg4 was determined to a resolution of 3.2 Å and now provides the unprecedented ability of rational design of specific and potent GMT inhibitors. The x-ray crystal structure of Vrg4 was determined in the luminal-facing state in the complex with GDP-mannose [111] and the presence of separate pockets for guanidine and mannose recognition explains how this family distinguishes between different substrates. An important conserved ‘GALNK’-motif is required for the recognition of the mannose group (Figure 6) [3,111]. Similarly, the ‘FYNN’-motif found in various species is involved in the binding of the guanine moiety of GDP-Man [111]. Therefore, amino acid substitution in these motives alters the Man content of the fungal cell.

*C. neoformans* possess two GMTs, GMT1, and GMT2 [36,69,117,119]. A mutant deficient in GMT1 shows defects in the capsule biosynthesis, which produces a smaller capsule [36,69]. Double mutant cells of both GMT1 and GMT2 were viable but with a reduced ability in mannosylation and capsule synthesis [113]. In mouse models infected with *Cryptococcus*, the GMT1/GMT2 double mutant was avirulent [113]. In addition, while both GMT1 and GMT2 are needed for the translocation of GDP-Man, their expression differs. GMT2 expression increases when there is an increased need for Man due to external triggers or stimuli [119]. Likewise, two GMTs, *gmtA* and *gmtB* in *A*. *nidulans*, function in a similar manner but at different expression levels [119], which highlights the significance of GMT as a potential drug target. This is particularly the case given that the GMT is absent in humans and has an overall low sequence identity (29%) to the GDP-Fucose transporter (GFT, SLC35C1), with the GALNK-motif replaced by a GTAKA-motif in the GFT (Figure 6).

### 7.2. UDP-Galactofuranose Transporter (UGfT)

As previously mentioned, Gal*f* plays an important role in the biosynthesis of galactomannan, *N*-glycans, *O*-glycans, and glycosphingolipids [67,80,86]. The deletion of UG*f*T (*glfB* or *ugtA*) in *Aspergillus* strains had a significant effect on their cell wall structure, with a complete absence of Gal, which exposed the Man backbone [86,120] and impaired cell growth [42,67,86,121]. Only the UG*f*T, and not the human UDP-Gal transporter (UGT), recognizes UDP-Gal*f* [121]. The effect of Gal*f* absence on fungal virulence was assessed using different *A. fumigatus* strains deficient in UGM with contradicting results [86,120]. Deletion of the gene encoding UGM was associated with a reduced growth and sporulation, a thinner cell wall, increased sensitivity to antifungals, and reduced virulence in mice [86]. In contrast, Lamarre et al. did not observe any effect on the virulence due to Gal*f* absence but reported an increased adhesion of the cell wall likely due to an increase in galactosaminogalactan [120]. A UGM deficient strain of *C. neoformans* showed a minimal effect on the infection in mouse [122]. Similar to the potential of targeting the Man component of galactomannan by inhibiting the GMT, targeting UDP-Gal*f* biosynthesis, which is also not present in humans, may provide the potential for the development of adjunct therapeutics. Further studies are, however, needed to fully assess the impact Gal*f*-absence and its effect as an immunogenic in humans.

### 7.3. UDP-Glucuronic Acid Transporter (UGlcAT)

UDP-Glucuronic acid transporter (UGlcAT) is of particular importance in the *Cryptococcus* genera since GlcA is a constituent of GXM and GXMGal, which are important components of the polysaccharide capsule [37]. *C. neoformans* has a single UGlcAT, Uut1 that is specific for UDP-GlcA [37]. The disruption of this transporter through knockdown of *uut1* in *C. neoformans* resulted in a complete absence of the cell capsule, which increased susceptibility to external stress [37]. The null mutant of *uut1* also resulted in the accumulation of UDP-Glc due to a regulatory pathway controlling the biosynthesis of UDP-GlcA and UDP-Xyl [37]. The *uut1* mutants have up to 4-fold higher UDP-Glc than UDP-GlcA levels compared to the wild type [37]. Further validation of the importance of UDP-GlcA originates from studies of UDP-glucose dehydrogenase (Ugd1), which converts UDP-Glc to UDP-GlcA (see Figure 3 for structures). A *Ugd1* mutant showed complete loss of UDP-GlcA in *C. neoformans* [123], and a reduction in the integrity of the cell wall and capsule, which makes the fungal cell thermo-sensitive and sensitive to external stress factors [37,123,124]. Taken together, these studies not only show the importance of UDP-GlcA in the biosynthesis of the capsule but also show the requirement of the corresponding NST for fungal cell biosynthesis and virulence. 

### 7.4. UDP-Xylose Transporter (UXT)

Xylose (Xyl) is a monosaccharide that forms an integral part of the capsule in the *Cryptococcus* genera, with 40–60% of GXM and a small percentage of GXMGal containing Xyl and GlcA residues [92,124]. Since UDP-Xyl is dependent on the biosynthesis of the precursor, UDP-GlcA mutations that result in the absence of UDP-GlcA will also result in a lack of UDP-Xyl [123]. Two known UXTs, called Uxt1 and Uxt2, are both responsible for the transport of UDP-Xyl in *C. neoformans*. Uxt1, which is the major UDP-xylose transporter, is constitutively expressed and localized in the Golgi apparatus whereas uxt2 expression increased during capsule induction and was found in the endoplasmic reticulum [92]. Whereas deletion of both *uxt1* and *uxt2* resulted in a complete absence of Xyl, deletion of *uxt1* alone caused a reduction in the level of Xyl residues in GXM [92]. There was no clear effect observed in the *uxt2* deletion mutant [92], which indicates that Uxt1 and Uxt2 have distinct but redundant functions. Deficiency of the two *C. neoformans* UXTs, Uxt1 and Uxt2, caused an absence of Xyl in GXM, which led to a thinner capsule and changes the cell capsule morphology [92]. This resulted in an increase in immune detection and clearance of these pathogens in mice [92]. Absence of Uxt1 or Uxt2 alone did not affect virulence.

## 8. Conclusions

Invasive fungal infections are an ever-increasing problem in today’s society due to the growing immunocompromised population. There are a variety of antifungal treatment classes that target different aspects of the fungal cell biosynthesis. With the high prevalence of IFI cases worldwide projected to increase over the next decade and the progression of drug resistant fungal strains, novel antifungal treatments are urgently needed. Development of new antifungals is challenging due to the limited number of targets that differ between humans and pathogenic fungi [1,25]. In order to develop effective novel therapeutics, target selectivity for more accurate and safe treatments is essential. The knowledge of the biosynthesis, growth, and virulence in fungi is vital not only to understand their pathogenic mechanisms but also to identify potential antifungal targets. Numerous enzymes and transport proteins are involved in the biosynthesis of the fungal cell wall and are, therefore, attractive targets for drug discovery. In the current article, we have highlighted the potential of several nucleotide sugar transporters (NSTs) as targets to develop novel antifungal treatments. The GDP-Man transporter is particularly attractive as a prospective target for antifungal drug development due to its absence in humans and its proven importance for the growth of *Aspergillus*, *Candida,* and *Cryptococcus*. The recent elucidation of the x-ray crystal structure of several NSTs paves the way for the rational design of specific NSTs inhibitors.
